# Glances at the Hospitals

**Published:** 1902-07-19

**Authors:** 


### GLANCES AT THE HOSPITALS.

### DERBYSHIRE ROYAL INFIRMARY.

The total ordinary income was £8,230, and the ordinary expenditure was £9,501, showing a deficiency of £1,270. The extraordinary receipts, including a donation of £1,000 from Mr. J. Marshall, amounted to £1,325, and the extraordinary expenditure to £393. A good arrangement has been entered into with the Derby Corporation. The hospital has paid over a sum of £2,000, and the corporation has agreed to provide accommodation, in the Borough Infectious Hospital, for such scarlet fever cases as may be sent from the infirmary. In addition to this arrangement, the infirmary committee have decided to spend £4,300 in providing an isolation block for the treatment of septic cases. At the beginning of the year the infirmary contained 147 patients, and 1,895 were admitted during the year, making a total of 2,042. Of these, 1,324 were discharged cured, 277 relieved, 141 were discharged for various reasons, 150 died, and 150 remained under treatment. The total number of out-patients was 8,402. The medical and surgical tables are drawn out with care, and are provided with an extra column for "remarks." Much may be learned from these. There were 95 cases of enteric fever. Of these 86 were cured, and 9 died. The immediate causes of death being toxæmia in 1, hæmorrhage in 3, perforation in 1, bronchitis in 1, bronchopneumonia in 2, and meningitis in 1. There were 20 cases of diphtheria. Of these 18 were cured, 1 relieved, 1 died. Of the total, tracheotomy was performed in 7 instances. Acute rheumatism was represented by 43 cases, all of whom recovered, and we learn that 14 were complicated by endocarditis, 4 by pericarditis, 5 by pleurisy, 1 by meningitis, 1 by epistaxis, and 1 by purpura. Herniotomy was performed 45 times, with 4 deaths. Appendectomy 6 times, all being successful. There was 1 case of perforated typhoid ulcer, and death took place from pneumonia 22 days after the operation. A total of 163 operations were performed in the eye department, and all were followed by recovery. Anæsthetics were administered 1,102 times; ether being the agent in 478, chloroform in 366, a mixture of chloroform and ether in 45, nitrous oxide in 106, nitrous oxide and ether in 3, and local anæsthesia was produced by cocaine in 104 cases. There was 1 death, a whooping-cough case, aged 9 months, to whom a mixture of chloroform and ether had been given. Tables compiled in this way are really useful for purposes of comparison, and there are many infirmaries which could hardly do better than copy the Derby system of medical and surgical statistics. We suggest, however, that the secretary should compile a tabulated statement of the cost per head per week, the cost per bed occupied, the cost per patient, the average stay of each patient in the hospital, and the percentage of deaths.

ROYAL HANTS COUNTY HOSPITAL.

THE total ordinary receipts were £5,459, and the total ordinary expenditure was £6,216, showing a deficit of £757 on the year's working. It would seem that the average number of in-patients has increased from 73 to 86, and that the average cost per bed occupied has decreased from £70 to £64, and further that the average cost of each in-patient has been reduced from £7 2s. 6d. to £5. The total income from all sources was £6,673, and the total expenditure was £9,550, the latter exceeding the former by £2,877. The most of the extraordinary expenditure was incurred by draining the isolation block, and by the new system of heating the hospital, consequently these will not occur again. Investments to the extent of £2,500 were sold, and a county meeting was held at which donations to the amount of £3,600 were announced. At the beginning of the year there were 105 patients in residence, 983 were admitted during the year, making a total of 1,088 under treatment. Of these 522 were discharged recovered, 254 much improved, 103 improved, 64 for various reasons, 54 died, and 91 remained in the hospital. The medical and surgical tables merely tell us that so many patients were treated for so many diseases, and not even the operations table gives results, nor is there any table showing the causes of death.

ROYAL BERKSHIRE HOSPITAL.

THE total ordinary income was £7,845, and the total ordinary expenditure was £9,800, being close on £2,000 above the income. Previous to this there was a balance of £3,355 due to the treasurer. To meet this deficit, Indian Railway Stock to the value of £2,400 has been sold, legacies to the extent of £570 have been received, and £600 from the profits arising from the Private Nurses' Fund have been taken. The private nursing staff has been reorganised. The salaries have been raised to £35, increased £5 a year to £45, and it is proposed to take a separate house for this staff. The hospital contained 128 patients at the beginning of the year, 1,539 were admitted, making a total for the year of 1,667. There were 2,805 out-patients. Of the in-patients we notice that 757 were discharged cured, 282 relieved, 285 made out-patients, not relieved 61; for other reasons, 127 died and 144 remained under treatment. Like so many other hospitals, the medical and surgical statistics are incomplete. The total number of operations performed during the year was 1,561. Of these 127 were for abdominal diseases, and it would have been interesting and perhaps useful to have the details of many of these cases. There is a table showing the cause of death in the 127 in-patients. No anæsthetic table is given.

